# Supplementary material for: End Sequence Analysis Toolkit (ESAT) expands the extractable information from single-cell RNA-seq data
Source: Genome Res. 2016 Oct;26(10):1397–410. doi: 10.1101/gr.207902.116 (PMC5052061; doi:10.1101/gr.207902.116)
Supplement: Supplemental Material [file supp_gr.207902.116_Supp_Materials.tar.gz › ESAT-esat_v0.1/doc/html/classbroad_1_1core_1_1datastructures_1_1_matrix_with_headers.html]

ESAT: broad.core.datastructures.MatrixWithHeaders Class Reference


ESAT


- Main Page
- Related Pages
- Packages
- Classes
- Files

- Class List
- Class Index
- Class Hierarchy
- Class Members

All Classes Namespaces Files Functions Variables Enumerator Pages

- broad
- core
- datastructures
- MatrixWithHeaders

Public Member Functions |
Protected Member Functions |
List of all members

broad.core.datastructures.MatrixWithHeaders Class Reference

Inheritance diagram for broad.core.datastructures.MatrixWithHeaders:

[legend]

|  |  |
| --- | --- |
| Public Member Functions | |
|  | MatrixWithHeaders (String inputFile) throws IOException, ParseException |
|  | |
|  | MatrixWithHeaders (BufferedReader br) throws IOException, ParseException |
|  | |
|  | MatrixWithHeaders (Matrix data, List< String > rowNames, List< String > colNames) |
|  | |
|  | MatrixWithHeaders (Matrix data, List< String > rowNames, List< String > colNames, List< String > rowDescriptions) |
|  | |
|  | MatrixWithHeaders (List< String > rows, List< String > columns, Map< String, String > pidToName) |
|  | |
|  | MatrixWithHeaders (MatrixWithHeaders matrix, List< String > rows, List< String > columns) |
|  | |
|  | MatrixWithHeaders (List< String > rows, List< String > columns) |
|  | |
|  | MatrixWithHeaders (List< String > rows, List< String > columns, List< String > rowDescriptions) |
|  | |
|  | MatrixWithHeaders (String gctFile, String chipFile) throws IOException, ParseException |
|  | |
|  | MatrixWithHeaders (List< List< Double >> rawData, ArrayList< String > rowNameList, List< String > columnNameList) |
|  | |
|  | MatrixWithHeaders (ArrayList< String > rows, ArrayList< String > columns, int DefMatVal) |
|  | |
| Map< String, String > | getPIDToName () |
|  | |
| void | write (BufferedWriter bw) throws IOException |
|  | |
| void | write (String fileName) throws IOException |
|  | |
| void | writeGCT (BufferedWriter bw) throws IOException |
|  | |
| void | writeGCT (String save) throws IOException |
|  | |
| void | writeGCT (String save, Collection< String > columns) throws IOException |
|  | |
| void | writeGCT (String save, Map< String, Collection< String >> experimentInfo, boolean ordered) throws IOException |
|  | |
| void | writeGCT (String save, Map< String, Collection< String >> experimentInfo, Map< String, ExpressionExperimentInfo > fullInfo, boolean info) throws IOException |
|  | |
| void | writeGCT (String save, Collection< String > columns, Collection< String > rows) throws IOException |
|  | |
| void | writeGCTWithHeaders (String save, Map< String, String > columnMapping) throws IOException |
|  | |
| void | writeGCT (String save, Collection< String > columns, Collection< String > rows, int precision) throws IOException |
|  | |
| void | writeGCT (String save, Map< String, String > columns, Map< String, String > rows) throws IOException |
|  | |
| void | writeGCT (String save, Map< String, String > columns, Collection< String > rows) throws IOException |
|  | |
| void | writeGCT (String save, Map< String, String > columns) throws IOException |
|  | |
| void | writeGCT (String save, File chipFile) throws IOException |
|  | |
| Matrix | dataInverse () |
|  | |
| int | columnDimension () |
|  | |
| int | rowDimension () |
|  | |
| double | get (int i, int j) |
|  | |
| double[] | getColumn (int j) |
|  | |
| double[] | getColumn (String columnName) |
|  | |
| double[] | getRow (String rowName) |
|  | |
| void | set (int i, int j, double val) |
|  | |
| void | set (String row, String column, double value) |
|  | |
| void | setRow (String row, double[] vals) |
|  | |
| void | setColumn (double[] vals, String column) |
|  | |
| void | setColumn (double[] vals, int column) |
|  | |
| void | set (String row, int colIdx, double value) |
|  | |
| void | set (int rowIdx, String column, double value) |
|  | |
| double | get (String row, String column) |
|  | |
| boolean | containsColumn (String colName) |
|  | |
| boolean | containsRow (String rowName) |
|  | |
| double | get (String rowName, int colIdx) |
|  | |
| double | get (int rowIdx, String colName) |
|  | |
| List< String > | getColumnNames () |
|  | |
| List< String > | getRowNames () |
|  | |
| String | getRowName (int i) |
|  | |
| String | getColoumnName (int i) |
|  | |
| List< String > | getRowDescriptions () |
|  | |
| boolean | hasColumn (String column) |
|  | |
| boolean | hasRow (String row) |
|  | |
| List< Integer > | getIndecesForRowDescription (String rowDescription) |
|  | |
| void | setRowDescription (int rowIdx, String rowDescription) |
|  | |
| void | setRowDescription (String row, String rowDescription) |
|  | |
| MatrixWithHeaders | filterValuesLargerThanUsingColumn (int colIdx, double maxValue) |
|  | |
| MatrixWithHeaders | filterValuesSmallerThanUsingColumn (int colIdx, double minValue) |
|  | |
| MatrixWithHeaders | filterValuesRangeUsingColumn (int colIdx, double minVal, double maxVal) |
|  | |
| boolean | isColumnNonTrivial (String colName) |
|  | |
| void | compressMatrixByRowDescriptions () |
|  | |
| void | normalizeByMean () |
|  | |
| void | zColumnNormalize () |
|  | |
| void | zRowNormalize () |
|  | |
| void | normalizeBy (double constant) |
|  | |
| void | normalizeColumnsByMean () |
|  | |
| void | normalizeColumnsByMedian () |
|  | |
| MatrixWithHeaders | submatrixByRowDescriptions (Collection< String > rowDescriptions, List< String > newRowNames) |
|  | |
| MatrixWithHeaders | submatrixByRowNames (Collection< String > rowNames) |
|  | |
| MatrixWithHeaders | submatrixByRowNames (String rowName) |
|  | |
| MatrixWithHeaders | submatrixByRowNames (String[] rowNames) |
|  | |
| MatrixWithHeaders | submatrixByColumnNames (Collection< String > columnNames) |
|  | |
| MatrixWithHeaders | submatrixByColumnIndex (Collection< Integer > columnIndex) |
|  | |
| MatrixWithHeaders | submatrixByColumnNames (String[] columnNames) |
|  | |
| MatrixWithHeaders | zScoresByColumn (List< String > controlRows) |
|  | |
| MatrixWithHeaders | tScoresByRow (List< Integer > controlRows, List< Integer > sampleRows, String comparisonName, double fudgeFactor) |
|  | |
| MatrixWithHeaders | rank () |
|  | |
| void | quantileNormalizeColumns () |
|  | |
| MatrixWithHeaders | confidenceByColumn (List< String > controlRows) |
|  | |
| MatrixWithHeaders | sortList (int columnIndex) |
|  | |
| void | append (MatrixWithHeaders other) |
|  | |
| void | appendColumns (MatrixWithHeaders other) |
|  | |
| void | appendRows (MatrixWithHeaders other) |
|  | |
| void | add (MatrixWithHeaders m2) |
|  | |
| void | log2 () |
|  | |
| void | log2 (double fudge) |
|  | |
| void | log10 () |
|  | |
| void | log10 (double fudge) |
|  | |
| void | pow () |
|  | |
| void | round () |
|  | |
| MatrixWithHeaders | times (MatrixWithHeaders other) |
|  | |
| void | minus (MatrixWithHeaders other) |
|  | |
| Matrix | getData () |
|  | |
| Map< String, Collection  < Integer > > | getNanostringReplicateMap () |
|  | |
| Map< String, String > | getNanostringProbeClasses () |
|  | |
| int | getNumberColumns () |
|  | |
| int | getNumberRows () |
|  | |
| Map< String, double[]> | toMap () |
|  | |
| void | writeGMT (String save, double fold) throws IOException |
|  | |
| MatrixWithHeaders | quantileNormalize () |
|  | |
| MatrixWithHeaders | copy () |
|  | |
| MatrixWithHeaders | medianCenterColumns () |
|  | |
| MatrixWithHeaders | medianCenterRows () |
|  | |
| void | medianCenterColumnsThis () |
|  | |
| void | medianCenterRowsThis () |
|  | |
| void | meanNormalizeColumns () throws IllegalStateException |
|  | |
| void | medianNormalizeColumns () throws IllegalStateException |
|  | |
| void | meanNormalizeRows () throws IllegalStateException |
|  | |
| void | medianNormalizeRows () throws IllegalStateException |
|  | |
| MatrixWithHeaders | scaleNorm () |
|  | |
| void | columnDensityNormalization () throws IllegalStateException |
|  | |
| void | rowDensityNormalization () throws IllegalStateException |
|  | |
| void | setPIDToName (Map< String, String > rowDescriptions) |
|  | |
| void | writeCLS (String string, Collection< String > subset, Collection< String > negatives) throws IOException |
|  | |
| void | writeGCTAndCLS (String save, Map< String, ExpressionExperimentInfo > experimentInfo) throws IOException |
|  | |
| void | randomPermuteColumns () |
|  | |
| void | addRow (String row, String description, double[] vals) |
|  | |
| void | addColumn (String column) |
|  | |
| MatrixWithHeaders | filterInvariantGenes (MatrixWithHeaders data, double fold) |
|  | |
| void | writeBox (String save) throws IOException |
|  | |
| MatrixWithHeaders | excludeByRowNames (Collection< String > flaggedGenes) |
|  | |
| double[] | getRow (int i) |
|  | |
| void | floor (double floor) |
|  | |
| void | writeRowNamesToFile (String outFile) throws IOException |
|  | |
| void | writeColoumnNamesToFile (String outFile) throws IOException |
|  | |
| double[] | getMedianOverAllRows () |
|  | |
| double[] | getMeanOverAllRows () |
|  | |
| double[] | getRow (String gene, Collection< String > group1) |
|  | |
| double[] | getValues (String gene, Collection< String > controls) |
|  | |
| boolean | hasNanostringProbeClasses () |
|  | |
| MatrixWithHeaders | multiplyColumnsWithConstants (double[] constants) |
|  | |
| MatrixWithHeaders | multiplyColumnsWithConstants (Map< String, Double > constants) |
|  | |

|  |  |
| --- | --- |
| Protected Member Functions | |
|  | MatrixWithHeaders () |
|  | |
| void | initFromReader (BufferedReader br) throws IOException, ParseException |
|  | |
| void | initFromRegularMatrix (BufferedReader br, String header) throws IOException, ParseException |
|  | |
| void | initGCTFromReader (BufferedReader br) throws IOException, ParseException |
|  | |
| void | initFromNanostring (BufferedReader br, String header) throws IOException, ParseException |
|  | |
| void | initNameIndexMaps (List< String > rowNameList, List< String > columnNameList) |
|  | |
| void | setData (Matrix data) |
|  | |
| void | initRowIndexMaps (List< String > rowNameList) |
|  | |
| void | initColIndexMaps (List< String > columnNameList) |
|  | |
| void | initRowDescrIndexMaps (List< String > rowDescriptionList) |
|  | |

## Constructor & Destructor Documentation

|  |  |  |  |  |
| --- | --- | --- | --- | --- |
| broad.core.datastructures.MatrixWithHeaders.MatrixWithHeaders | ( |  | ) |  |

protected

Here is the caller graph for this function:

|  |  |  |  |  |  |
| --- | --- | --- | --- | --- | --- |
| broad.core.datastructures.MatrixWithHeaders.MatrixWithHeaders | ( | String | *inputFile* | ) | throws IOException, ParseException |

Here is the call graph for this function:

|  |  |  |  |  |  |
| --- | --- | --- | --- | --- | --- |
| broad.core.datastructures.MatrixWithHeaders.MatrixWithHeaders | ( | BufferedReader | *br* | ) | throws IOException, ParseException |

Here is the call graph for this function:

|  |  |  |  |
| --- | --- | --- | --- |
| broad.core.datastructures.MatrixWithHeaders.MatrixWithHeaders | ( | Matrix | *data*, |
|  |  | List< String > | *rowNames*, |
|  |  | List< String > | *colNames* |
|  | ) |  |  |

Here is the call graph for this function:

|  |  |  |  |
| --- | --- | --- | --- |
| broad.core.datastructures.MatrixWithHeaders.MatrixWithHeaders | ( | Matrix | *data*, |
|  |  | List< String > | *rowNames*, |
|  |  | List< String > | *colNames*, |
|  |  | List< String > | *rowDescriptions* |
|  | ) |  |  |

Here is the call graph for this function:

|  |  |  |  |
| --- | --- | --- | --- |
| broad.core.datastructures.MatrixWithHeaders.MatrixWithHeaders | ( | List< String > | *rows*, |
|  |  | List< String > | *columns*, |
|  |  | Map< String, String > | *pidToName* |
|  | ) |  |  |

Here is the call graph for this function:

|  |  |  |  |
| --- | --- | --- | --- |
| broad.core.datastructures.MatrixWithHeaders.MatrixWithHeaders | ( | MatrixWithHeaders | *matrix*, |
|  |  | List< String > | *rows*, |
|  |  | List< String > | *columns* |
|  | ) |  |  |

Create a new matrix that has a subset of columns and rows of the original matrix

Parameters
:   |  |  |
    | --- | --- |
    | matrix |  |
    | colsInRows |  |
    | colsInRows2 |  |

|  |  |  |  |
| --- | --- | --- | --- |
| broad.core.datastructures.MatrixWithHeaders.MatrixWithHeaders | ( | List< String > | *rows*, |
|  |  | List< String > | *columns* |
|  | ) |  |  |

Here is the call graph for this function:

|  |  |  |  |
| --- | --- | --- | --- |
| broad.core.datastructures.MatrixWithHeaders.MatrixWithHeaders | ( | List< String > | *rows*, |
|  |  | List< String > | *columns*, |
|  |  | List< String > | *rowDescriptions* |
|  | ) |  |  |

Here is the call graph for this function:

|  |  |  |  |
| --- | --- | --- | --- |
| broad.core.datastructures.MatrixWithHeaders.MatrixWithHeaders | ( | String | *gctFile*, |
|  |  | String | *chipFile* |
|  | ) |  | throws IOException, ParseException |

Here is the call graph for this function:

|  |  |  |  |
| --- | --- | --- | --- |
| broad.core.datastructures.MatrixWithHeaders.MatrixWithHeaders | ( | List< List< Double >> | *rawData*, |
|  |  | ArrayList< String > | *rowNameList*, |
|  |  | List< String > | *columnNameList* |
|  | ) |  |  |

|  |  |  |  |
| --- | --- | --- | --- |
| broad.core.datastructures.MatrixWithHeaders.MatrixWithHeaders | ( | ArrayList< String > | *rows*, |
|  |  | ArrayList< String > | *columns*, |
|  |  | int | *DefMatVal* |
|  | ) |  |  |

## Member Function Documentation

|  |  |  |  |  |  |
| --- | --- | --- | --- | --- | --- |
| void broad.core.datastructures.MatrixWithHeaders.add | ( | MatrixWithHeaders | *m2* | ) |  |

Here is the caller graph for this function:

|  |  |  |  |  |  |
| --- | --- | --- | --- | --- | --- |
| void broad.core.datastructures.MatrixWithHeaders.addColumn | ( | String | *column* | ) |  |

Here is the call graph for this function:

|  |  |  |  |
| --- | --- | --- | --- |
| void broad.core.datastructures.MatrixWithHeaders.addRow | ( | String | *row*, |
|  |  | String | *description*, |
|  |  | double[] | *vals* |
|  | ) |  |  |

Here is the call graph for this function:

|  |  |  |  |  |  |
| --- | --- | --- | --- | --- | --- |
| void broad.core.datastructures.MatrixWithHeaders.append | ( | MatrixWithHeaders | *other* | ) |  |

Here is the call graph for this function:

Here is the caller graph for this function:

|  |  |  |  |  |  |
| --- | --- | --- | --- | --- | --- |
| void broad.core.datastructures.MatrixWithHeaders.appendColumns | ( | MatrixWithHeaders | *other* | ) |  |

Here is the call graph for this function:

|  |  |  |  |  |  |
| --- | --- | --- | --- | --- | --- |
| void broad.core.datastructures.MatrixWithHeaders.appendRows | ( | MatrixWithHeaders | *other* | ) |  |

Here is the call graph for this function:

|  |  |  |  |  |
| --- | --- | --- | --- | --- |
| void broad.core.datastructures.MatrixWithHeaders.columnDensityNormalization | ( |  | ) | throws IllegalStateException |

Normalizes each column so it adds up to 1

Exceptions
:   |  |  |
    | --- | --- |
    | IllegalStateException | if a column contains negative numbers |

|  |  |  |  |  |
| --- | --- | --- | --- | --- |
| int broad.core.datastructures.MatrixWithHeaders.columnDimension | ( |  | ) |  |

Here is the caller graph for this function:

|  |  |  |  |  |
| --- | --- | --- | --- | --- |
| void broad.core.datastructures.MatrixWithHeaders.compressMatrixByRowDescriptions | ( |  | ) |  |

Replaces each set of rows with same description with a row with column values the mean of the original row column values

Here is the call graph for this function:

|  |  |  |  |  |  |
| --- | --- | --- | --- | --- | --- |
| MatrixWithHeaders broad.core.datastructures.MatrixWithHeaders.confidenceByColumn | ( | List< String > | *controlRows* | ) |  |

Here is the call graph for this function:

|  |  |  |  |  |  |
| --- | --- | --- | --- | --- | --- |
| boolean broad.core.datastructures.MatrixWithHeaders.containsColumn | ( | String | *colName* | ) |  |

|  |  |  |  |  |  |
| --- | --- | --- | --- | --- | --- |
| boolean broad.core.datastructures.MatrixWithHeaders.containsRow | ( | String | *rowName* | ) |  |

|  |  |  |  |  |
| --- | --- | --- | --- | --- |
| MatrixWithHeaders broad.core.datastructures.MatrixWithHeaders.copy | ( |  | ) |  |

Here is the call graph for this function:

Here is the caller graph for this function:

|  |  |  |  |  |
| --- | --- | --- | --- | --- |
| Matrix broad.core.datastructures.MatrixWithHeaders.dataInverse | ( |  | ) |  |

Takes the inverse (or pseudo inverse if the underlying data is not a squared matrix)

Returns

|  |  |  |  |  |  |
| --- | --- | --- | --- | --- | --- |
| MatrixWithHeaders broad.core.datastructures.MatrixWithHeaders.excludeByRowNames | ( | Collection< String > | *flaggedGenes* | ) |  |

Here is the call graph for this function:

|  |  |  |  |
| --- | --- | --- | --- |
| MatrixWithHeaders broad.core.datastructures.MatrixWithHeaders.filterInvariantGenes | ( | MatrixWithHeaders | *data*, |
|  |  | double | *fold* |
|  | ) |  |  |

Here is the call graph for this function:

|  |  |  |  |
| --- | --- | --- | --- |
| MatrixWithHeaders broad.core.datastructures.MatrixWithHeaders.filterValuesLargerThanUsingColumn | ( | int | *colIdx*, |
|  |  | double | *maxValue* |
|  | ) |  |  |

Here is the call graph for this function:

|  |  |  |  |
| --- | --- | --- | --- |
| MatrixWithHeaders broad.core.datastructures.MatrixWithHeaders.filterValuesRangeUsingColumn | ( | int | *colIdx*, |
|  |  | double | *minVal*, |
|  |  | double | *maxVal* |
|  | ) |  |  |

Here is the call graph for this function:

|  |  |  |  |
| --- | --- | --- | --- |
| MatrixWithHeaders broad.core.datastructures.MatrixWithHeaders.filterValuesSmallerThanUsingColumn | ( | int | *colIdx*, |
|  |  | double | *minValue* |
|  | ) |  |  |

Here is the call graph for this function:

|  |  |  |  |  |  |
| --- | --- | --- | --- | --- | --- |
| void broad.core.datastructures.MatrixWithHeaders.floor | ( | double | *floor* | ) |  |

Resets all values below floor to floor.

Parameters
:   |  |  |
    | --- | --- |
    | floor |  |

Here is the call graph for this function:

|  |  |  |  |
| --- | --- | --- | --- |
| double broad.core.datastructures.MatrixWithHeaders.get | ( | int | *i*, |
|  |  | int | *j* |
|  | ) |  |  |

Here is the caller graph for this function:

|  |  |  |  |
| --- | --- | --- | --- |
| double broad.core.datastructures.MatrixWithHeaders.get | ( | String | *row*, |
|  |  | String | *column* |
|  | ) |  |  |

|  |  |  |  |
| --- | --- | --- | --- |
| double broad.core.datastructures.MatrixWithHeaders.get | ( | String | *rowName*, |
|  |  | int | *colIdx* |
|  | ) |  |  |

|  |  |  |  |
| --- | --- | --- | --- |
| double broad.core.datastructures.MatrixWithHeaders.get | ( | int | *rowIdx*, |
|  |  | String | *colName* |
|  | ) |  |  |

|  |  |  |  |  |  |
| --- | --- | --- | --- | --- | --- |
| String broad.core.datastructures.MatrixWithHeaders.getColoumnName | ( | int | *i* | ) |  |

|  |  |  |  |  |  |
| --- | --- | --- | --- | --- | --- |
| double [] broad.core.datastructures.MatrixWithHeaders.getColumn | ( | int | *j* | ) |  |

Here is the call graph for this function:

Here is the caller graph for this function:

|  |  |  |  |  |  |
| --- | --- | --- | --- | --- | --- |
| double [] broad.core.datastructures.MatrixWithHeaders.getColumn | ( | String | *columnName* | ) |  |

|  |  |  |  |  |
| --- | --- | --- | --- | --- |
| List<String> broad.core.datastructures.MatrixWithHeaders.getColumnNames | ( |  | ) |  |

Here is the caller graph for this function:

|  |  |  |  |  |
| --- | --- | --- | --- | --- |
| Matrix broad.core.datastructures.MatrixWithHeaders.getData | ( |  | ) |  |

|  |  |  |  |  |  |
| --- | --- | --- | --- | --- | --- |
| List<Integer> broad.core.datastructures.MatrixWithHeaders.getIndecesForRowDescription | ( | String | *rowDescription* | ) |  |

Here is the caller graph for this function:

|  |  |  |  |  |
| --- | --- | --- | --- | --- |
| double [] broad.core.datastructures.MatrixWithHeaders.getMeanOverAllRows | ( |  | ) |  |

|  |  |  |  |  |
| --- | --- | --- | --- | --- |
| double [] broad.core.datastructures.MatrixWithHeaders.getMedianOverAllRows | ( |  | ) |  |

|  |  |  |  |  |
| --- | --- | --- | --- | --- |
| Map<String, String> broad.core.datastructures.MatrixWithHeaders.getNanostringProbeClasses | ( |  | ) |  |

Here is the caller graph for this function:

|  |  |  |  |  |
| --- | --- | --- | --- | --- |
| Map<String, Collection<Integer> > broad.core.datastructures.MatrixWithHeaders.getNanostringReplicateMap | ( |  | ) |  |

|  |  |  |  |  |
| --- | --- | --- | --- | --- |
| int broad.core.datastructures.MatrixWithHeaders.getNumberColumns | ( |  | ) |  |

|  |  |  |  |  |
| --- | --- | --- | --- | --- |
| int broad.core.datastructures.MatrixWithHeaders.getNumberRows | ( |  | ) |  |

|  |  |  |  |  |
| --- | --- | --- | --- | --- |
| Map<String, String> broad.core.datastructures.MatrixWithHeaders.getPIDToName | ( |  | ) |  |

|  |  |  |  |  |  |
| --- | --- | --- | --- | --- | --- |
| double [] broad.core.datastructures.MatrixWithHeaders.getRow | ( | String | *rowName* | ) |  |

Here is the caller graph for this function:

|  |  |  |  |  |  |
| --- | --- | --- | --- | --- | --- |
| double [] broad.core.datastructures.MatrixWithHeaders.getRow | ( | int | *i* | ) |  |

|  |  |  |  |
| --- | --- | --- | --- |
| double [] broad.core.datastructures.MatrixWithHeaders.getRow | ( | String | *gene*, |
|  |  | Collection< String > | *group1* |
|  | ) |  |  |

|  |  |  |  |  |
| --- | --- | --- | --- | --- |
| List<String> broad.core.datastructures.MatrixWithHeaders.getRowDescriptions | ( |  | ) |  |

Here is the caller graph for this function:

|  |  |  |  |  |  |
| --- | --- | --- | --- | --- | --- |
| String broad.core.datastructures.MatrixWithHeaders.getRowName | ( | int | *i* | ) |  |

|  |  |  |  |  |
| --- | --- | --- | --- | --- |
| List<String> broad.core.datastructures.MatrixWithHeaders.getRowNames | ( |  | ) |  |

Here is the caller graph for this function:

|  |  |  |  |
| --- | --- | --- | --- |
| double [] broad.core.datastructures.MatrixWithHeaders.getValues | ( | String | *gene*, |
|  |  | Collection< String > | *controls* |
|  | ) |  |  |

|  |  |  |  |  |  |
| --- | --- | --- | --- | --- | --- |
| boolean broad.core.datastructures.MatrixWithHeaders.hasColumn | ( | String | *column* | ) |  |

|  |  |  |  |  |
| --- | --- | --- | --- | --- |
| boolean broad.core.datastructures.MatrixWithHeaders.hasNanostringProbeClasses | ( |  | ) |  |

Here is the call graph for this function:

|  |  |  |  |  |  |
| --- | --- | --- | --- | --- | --- |
| boolean broad.core.datastructures.MatrixWithHeaders.hasRow | ( | String | *row* | ) |  |

|  |  |  |  |  |  |
| --- | --- | --- | --- | --- | --- |
| void broad.core.datastructures.MatrixWithHeaders.initColIndexMaps | ( | List< String > | *columnNameList* | ) |  |

protected

Here is the caller graph for this function:

|  |  |  |  |
| --- | --- | --- | --- |
| void broad.core.datastructures.MatrixWithHeaders.initFromNanostring | ( | BufferedReader | *br*, |
|  |  | String | *header* |
|  | ) |  | throws IOException, ParseException |

protected

Here is the caller graph for this function:

|  |  |  |  |  |  |
| --- | --- | --- | --- | --- | --- |
| void broad.core.datastructures.MatrixWithHeaders.initFromReader | ( | BufferedReader | *br* | ) | throws IOException, ParseException |

protected

Here is the call graph for this function:

Here is the caller graph for this function:

|  |  |  |  |
| --- | --- | --- | --- |
| void broad.core.datastructures.MatrixWithHeaders.initFromRegularMatrix | ( | BufferedReader | *br*, |
|  |  | String | *header* |
|  | ) |  | throws IOException, ParseException |

protected

Here is the caller graph for this function:

|  |  |  |  |  |  |
| --- | --- | --- | --- | --- | --- |
| void broad.core.datastructures.MatrixWithHeaders.initGCTFromReader | ( | BufferedReader | *br* | ) | throws IOException, ParseException |

protected

Here is the caller graph for this function:

|  |  |  |  |
| --- | --- | --- | --- |
| void broad.core.datastructures.MatrixWithHeaders.initNameIndexMaps | ( | List< String > | *rowNameList*, |
|  |  | List< String > | *columnNameList* |
|  | ) |  |  |

protected

Here is the caller graph for this function:

|  |  |  |  |  |  |
| --- | --- | --- | --- | --- | --- |
| void broad.core.datastructures.MatrixWithHeaders.initRowDescrIndexMaps | ( | List< String > | *rowDescriptionList* | ) |  |

protected

|  |  |  |  |  |  |
| --- | --- | --- | --- | --- | --- |
| void broad.core.datastructures.MatrixWithHeaders.initRowIndexMaps | ( | List< String > | *rowNameList* | ) |  |

protected

|  |  |  |  |  |  |
| --- | --- | --- | --- | --- | --- |
| boolean broad.core.datastructures.MatrixWithHeaders.isColumnNonTrivial | ( | String | *colName* | ) |  |

returns true if the column has a nonzero value

|  |  |  |  |  |
| --- | --- | --- | --- | --- |
| void broad.core.datastructures.MatrixWithHeaders.log10 | ( |  | ) |  |

|  |  |  |  |  |  |
| --- | --- | --- | --- | --- | --- |
| void broad.core.datastructures.MatrixWithHeaders.log10 | ( | double | *fudge* | ) |  |

|  |  |  |  |  |
| --- | --- | --- | --- | --- |
| void broad.core.datastructures.MatrixWithHeaders.log2 | ( |  | ) |  |

|  |  |  |  |  |  |
| --- | --- | --- | --- | --- | --- |
| void broad.core.datastructures.MatrixWithHeaders.log2 | ( | double | *fudge* | ) |  |

|  |  |  |  |  |
| --- | --- | --- | --- | --- |
| void broad.core.datastructures.MatrixWithHeaders.meanNormalizeColumns | ( |  | ) | throws IllegalStateException |

Substitutes each value in a column with the ratio of the value and the mean of column value Only applicable to positive value matrices.

|  |  |  |  |  |
| --- | --- | --- | --- | --- |
| void broad.core.datastructures.MatrixWithHeaders.meanNormalizeRows | ( |  | ) | throws IllegalStateException |

Substitutes each value in a row with the ratio of the value and the mean of row value Only applicable to positive value matrices.

|  |  |  |  |  |
| --- | --- | --- | --- | --- |
| MatrixWithHeaders broad.core.datastructures.MatrixWithHeaders.medianCenterColumns | ( |  | ) |  |

Here is the call graph for this function:

|  |  |  |  |  |
| --- | --- | --- | --- | --- |
| void broad.core.datastructures.MatrixWithHeaders.medianCenterColumnsThis | ( |  | ) |  |

Mean centers the columns of the current matrix rather than creating a new one.

|  |  |  |  |  |
| --- | --- | --- | --- | --- |
| MatrixWithHeaders broad.core.datastructures.MatrixWithHeaders.medianCenterRows | ( |  | ) |  |

Here is the call graph for this function:

|  |  |  |  |  |
| --- | --- | --- | --- | --- |
| void broad.core.datastructures.MatrixWithHeaders.medianCenterRowsThis | ( |  | ) |  |

Mean centers the rows of the current matrix rather than creating a new one.

|  |  |  |  |  |
| --- | --- | --- | --- | --- |
| void broad.core.datastructures.MatrixWithHeaders.medianNormalizeColumns | ( |  | ) | throws IllegalStateException |

Substitutes each value in a column with the ratio of the value and the mean of column value Only applicable to positive value matrices.

|  |  |  |  |  |
| --- | --- | --- | --- | --- |
| void broad.core.datastructures.MatrixWithHeaders.medianNormalizeRows | ( |  | ) | throws IllegalStateException |

Substitutes each value in a row with the ratio of the value and the mean of row value Only applicable to positive value matrices.

|  |  |  |  |  |  |
| --- | --- | --- | --- | --- | --- |
| void broad.core.datastructures.MatrixWithHeaders.minus | ( | MatrixWithHeaders | *other* | ) |  |

Here is the call graph for this function:

|  |  |  |  |  |  |
| --- | --- | --- | --- | --- | --- |
| MatrixWithHeaders broad.core.datastructures.MatrixWithHeaders.multiplyColumnsWithConstants | ( | double[] | *constants* | ) |  |

Author
:   skadri Multiplies each column with a separate constant. That is, given a vector (dimension same as number of columns) multiplies all rows of column with same constant

Returns

Here is the call graph for this function:

|  |  |  |  |  |  |
| --- | --- | --- | --- | --- | --- |
| MatrixWithHeaders broad.core.datastructures.MatrixWithHeaders.multiplyColumnsWithConstants | ( | Map< String, Double > | *constants* | ) |  |

Author
:   skadri Multiplies each column with a separate constant. That is, given a vector (dimension same as number of columns) multiplies all rows of column with same constant

Returns

Here is the call graph for this function:

|  |  |  |  |  |  |
| --- | --- | --- | --- | --- | --- |
| void broad.core.datastructures.MatrixWithHeaders.normalizeBy | ( | double | *constant* | ) |  |

Here is the caller graph for this function:

|  |  |  |  |  |
| --- | --- | --- | --- | --- |
| void broad.core.datastructures.MatrixWithHeaders.normalizeByMean | ( |  | ) |  |

Here is the call graph for this function:

|  |  |  |  |  |
| --- | --- | --- | --- | --- |
| void broad.core.datastructures.MatrixWithHeaders.normalizeColumnsByMean | ( |  | ) |  |

Here is the call graph for this function:

|  |  |  |  |  |
| --- | --- | --- | --- | --- |
| void broad.core.datastructures.MatrixWithHeaders.normalizeColumnsByMedian | ( |  | ) |  |

Here is the call graph for this function:

|  |  |  |  |  |
| --- | --- | --- | --- | --- |
| void broad.core.datastructures.MatrixWithHeaders.pow | ( |  | ) |  |

|  |  |  |  |  |
| --- | --- | --- | --- | --- |
| MatrixWithHeaders broad.core.datastructures.MatrixWithHeaders.quantileNormalize | ( |  | ) |  |

Here is the call graph for this function:

|  |  |  |  |  |
| --- | --- | --- | --- | --- |
| void broad.core.datastructures.MatrixWithHeaders.quantileNormalizeColumns | ( |  | ) |  |

|  |  |  |  |  |
| --- | --- | --- | --- | --- |
| void broad.core.datastructures.MatrixWithHeaders.randomPermuteColumns | ( |  | ) |  |

|  |  |  |  |  |
| --- | --- | --- | --- | --- |
| MatrixWithHeaders broad.core.datastructures.MatrixWithHeaders.rank | ( |  | ) |  |

Here is the call graph for this function:

Here is the caller graph for this function:

|  |  |  |  |  |
| --- | --- | --- | --- | --- |
| void broad.core.datastructures.MatrixWithHeaders.round | ( |  | ) |  |

|  |  |  |  |  |
| --- | --- | --- | --- | --- |
| void broad.core.datastructures.MatrixWithHeaders.rowDensityNormalization | ( |  | ) | throws IllegalStateException |

Normalizes each row so it adds up to 1

Exceptions
:   |  |  |
    | --- | --- |
    | IllegalStateException | if a column contains negative numbers |

|  |  |  |  |  |
| --- | --- | --- | --- | --- |
| int broad.core.datastructures.MatrixWithHeaders.rowDimension | ( |  | ) |  |

Here is the caller graph for this function:

|  |  |  |  |  |
| --- | --- | --- | --- | --- |
| MatrixWithHeaders broad.core.datastructures.MatrixWithHeaders.scaleNorm | ( |  | ) |  |

Here is the call graph for this function:

|  |  |  |  |
| --- | --- | --- | --- |
| void broad.core.datastructures.MatrixWithHeaders.set | ( | int | *i*, |
|  |  | int | *j*, |
|  |  | double | *val* |
|  | ) |  |  |

Here is the caller graph for this function:

|  |  |  |  |
| --- | --- | --- | --- |
| void broad.core.datastructures.MatrixWithHeaders.set | ( | String | *row*, |
|  |  | String | *column*, |
|  |  | double | *value* |
|  | ) |  |  |

|  |  |  |  |
| --- | --- | --- | --- |
| void broad.core.datastructures.MatrixWithHeaders.set | ( | String | *row*, |
|  |  | int | *colIdx*, |
|  |  | double | *value* |
|  | ) |  |  |

|  |  |  |  |
| --- | --- | --- | --- |
| void broad.core.datastructures.MatrixWithHeaders.set | ( | int | *rowIdx*, |
|  |  | String | *column*, |
|  |  | double | *value* |
|  | ) |  |  |

|  |  |  |  |
| --- | --- | --- | --- |
| void broad.core.datastructures.MatrixWithHeaders.setColumn | ( | double[] | *vals*, |
|  |  | String | *column* |
|  | ) |  |  |

Here is the call graph for this function:

|  |  |  |  |
| --- | --- | --- | --- |
| void broad.core.datastructures.MatrixWithHeaders.setColumn | ( | double[] | *vals*, |
|  |  | int | *column* |
|  | ) |  |  |

Here is the call graph for this function:

|  |  |  |  |  |  |
| --- | --- | --- | --- | --- | --- |
| void broad.core.datastructures.MatrixWithHeaders.setData | ( | Matrix | *data* | ) |  |

protected

Here is the caller graph for this function:

|  |  |  |  |  |  |
| --- | --- | --- | --- | --- | --- |
| void broad.core.datastructures.MatrixWithHeaders.setPIDToName | ( | Map< String, String > | *rowDescriptions* | ) |  |

|  |  |  |  |
| --- | --- | --- | --- |
| void broad.core.datastructures.MatrixWithHeaders.setRow | ( | String | *row*, |
|  |  | double[] | *vals* |
|  | ) |  |  |

Here is the call graph for this function:

|  |  |  |  |
| --- | --- | --- | --- |
| void broad.core.datastructures.MatrixWithHeaders.setRowDescription | ( | int | *rowIdx*, |
|  |  | String | *rowDescription* |
|  | ) |  |  |

Here is the caller graph for this function:

|  |  |  |  |
| --- | --- | --- | --- |
| void broad.core.datastructures.MatrixWithHeaders.setRowDescription | ( | String | *row*, |
|  |  | String | *rowDescription* |
|  | ) |  |  |

Here is the call graph for this function:

|  |  |  |  |  |  |
| --- | --- | --- | --- | --- | --- |
| MatrixWithHeaders broad.core.datastructures.MatrixWithHeaders.sortList | ( | int | *columnIndex* | ) |  |

Here is the call graph for this function:

|  |  |  |  |  |  |
| --- | --- | --- | --- | --- | --- |
| MatrixWithHeaders broad.core.datastructures.MatrixWithHeaders.submatrixByColumnIndex | ( | Collection< Integer > | *columnIndex* | ) |  |

|  |  |  |  |  |  |
| --- | --- | --- | --- | --- | --- |
| MatrixWithHeaders broad.core.datastructures.MatrixWithHeaders.submatrixByColumnNames | ( | Collection< String > | *columnNames* | ) |  |

Here is the call graph for this function:

Here is the caller graph for this function:

|  |  |  |  |  |  |
| --- | --- | --- | --- | --- | --- |
| MatrixWithHeaders broad.core.datastructures.MatrixWithHeaders.submatrixByColumnNames | ( | String[] | *columnNames* | ) |  |

Here is the call graph for this function:

|  |  |  |  |
| --- | --- | --- | --- |
| MatrixWithHeaders broad.core.datastructures.MatrixWithHeaders.submatrixByRowDescriptions | ( | Collection< String > | *rowDescriptions*, |
|  |  | List< String > | *newRowNames* |
|  | ) |  |  |

Here is the call graph for this function:

|  |  |  |  |  |  |
| --- | --- | --- | --- | --- | --- |
| MatrixWithHeaders broad.core.datastructures.MatrixWithHeaders.submatrixByRowNames | ( | Collection< String > | *rowNames* | ) |  |

Here is the call graph for this function:

Here is the caller graph for this function:

|  |  |  |  |  |  |
| --- | --- | --- | --- | --- | --- |
| MatrixWithHeaders broad.core.datastructures.MatrixWithHeaders.submatrixByRowNames | ( | String | *rowName* | ) |  |

Here is the call graph for this function:

|  |  |  |  |  |  |
| --- | --- | --- | --- | --- | --- |
| MatrixWithHeaders broad.core.datastructures.MatrixWithHeaders.submatrixByRowNames | ( | String[] | *rowNames* | ) |  |

Here is the call graph for this function:

|  |  |  |  |  |  |
| --- | --- | --- | --- | --- | --- |
| MatrixWithHeaders broad.core.datastructures.MatrixWithHeaders.times | ( | MatrixWithHeaders | *other* | ) |  |

Here is the call graph for this function:

|  |  |  |  |  |
| --- | --- | --- | --- | --- |
| Map<String, double[]> broad.core.datastructures.MatrixWithHeaders.toMap | ( |  | ) |  |

Here is the call graph for this function:

|  |  |  |  |
| --- | --- | --- | --- |
| MatrixWithHeaders broad.core.datastructures.MatrixWithHeaders.tScoresByRow | ( | List< Integer > | *controlRows*, |
|  |  | List< Integer > | *sampleRows*, |
|  |  | String | *comparisonName*, |
|  |  | double | *fudgeFactor* |
|  | ) |  |  |

Here is the call graph for this function:

|  |  |  |  |  |  |
| --- | --- | --- | --- | --- | --- |
| void broad.core.datastructures.MatrixWithHeaders.write | ( | BufferedWriter | *bw* | ) | throws IOException |

Here is the caller graph for this function:

|  |  |  |  |  |  |
| --- | --- | --- | --- | --- | --- |
| void broad.core.datastructures.MatrixWithHeaders.write | ( | String | *fileName* | ) | throws IOException |

Here is the call graph for this function:

|  |  |  |  |  |  |
| --- | --- | --- | --- | --- | --- |
| void broad.core.datastructures.MatrixWithHeaders.writeBox | ( | String | *save* | ) | throws IOException |

|  |  |  |  |
| --- | --- | --- | --- |
| void broad.core.datastructures.MatrixWithHeaders.writeCLS | ( | String | *string*, |
|  |  | Collection< String > | *subset*, |
|  |  | Collection< String > | *negatives* |
|  | ) |  | throws IOException |

|  |  |  |  |  |  |
| --- | --- | --- | --- | --- | --- |
| void broad.core.datastructures.MatrixWithHeaders.writeColoumnNamesToFile | ( | String | *outFile* | ) | throws IOException |

|  |  |  |  |  |  |
| --- | --- | --- | --- | --- | --- |
| void broad.core.datastructures.MatrixWithHeaders.writeGCT | ( | BufferedWriter | *bw* | ) | throws IOException |

Here is the call graph for this function:

Here is the caller graph for this function:

|  |  |  |  |  |  |
| --- | --- | --- | --- | --- | --- |
| void broad.core.datastructures.MatrixWithHeaders.writeGCT | ( | String | *save* | ) | throws IOException |

Here is the call graph for this function:

|  |  |  |  |
| --- | --- | --- | --- |
| void broad.core.datastructures.MatrixWithHeaders.writeGCT | ( | String | *save*, |
|  |  | Collection< String > | *columns* |
|  | ) |  | throws IOException |

Here is the call graph for this function:

|  |  |  |  |
| --- | --- | --- | --- |
| void broad.core.datastructures.MatrixWithHeaders.writeGCT | ( | String | *save*, |
|  |  | Map< String, Collection< String >> | *experimentInfo*, |
|  |  | boolean | *ordered* |
|  | ) |  | throws IOException |

Here is the call graph for this function:

|  |  |  |  |
| --- | --- | --- | --- |
| void broad.core.datastructures.MatrixWithHeaders.writeGCT | ( | String | *save*, |
|  |  | Map< String, Collection< String >> | *experimentInfo*, |
|  |  | Map< String, ExpressionExperimentInfo > | *fullInfo*, |
|  |  | boolean | *info* |
|  | ) |  | throws IOException |

Here is the call graph for this function:

|  |  |  |  |
| --- | --- | --- | --- |
| void broad.core.datastructures.MatrixWithHeaders.writeGCT | ( | String | *save*, |
|  |  | Collection< String > | *columns*, |
|  |  | Collection< String > | *rows* |
|  | ) |  | throws IOException |

|  |  |  |  |
| --- | --- | --- | --- |
| void broad.core.datastructures.MatrixWithHeaders.writeGCT | ( | String | *save*, |
|  |  | Collection< String > | *columns*, |
|  |  | Collection< String > | *rows*, |
|  |  | int | *precision* |
|  | ) |  | throws IOException |

|  |  |  |  |
| --- | --- | --- | --- |
| void broad.core.datastructures.MatrixWithHeaders.writeGCT | ( | String | *save*, |
|  |  | Map< String, String > | *columns*, |
|  |  | Map< String, String > | *rows* |
|  | ) |  | throws IOException |

|  |  |  |  |
| --- | --- | --- | --- |
| void broad.core.datastructures.MatrixWithHeaders.writeGCT | ( | String | *save*, |
|  |  | Map< String, String > | *columns*, |
|  |  | Collection< String > | *rows* |
|  | ) |  | throws IOException |

|  |  |  |  |
| --- | --- | --- | --- |
| void broad.core.datastructures.MatrixWithHeaders.writeGCT | ( | String | *save*, |
|  |  | Map< String, String > | *columns* |
|  | ) |  | throws IOException |

Here is the call graph for this function:

|  |  |  |  |
| --- | --- | --- | --- |
| void broad.core.datastructures.MatrixWithHeaders.writeGCT | ( | String | *save*, |
|  |  | File | *chipFile* |
|  | ) |  | throws IOException |

Here is the call graph for this function:

|  |  |  |  |
| --- | --- | --- | --- |
| void broad.core.datastructures.MatrixWithHeaders.writeGCTAndCLS | ( | String | *save*, |
|  |  | Map< String, ExpressionExperimentInfo > | *experimentInfo* |
|  | ) |  | throws IOException |

Here is the call graph for this function:

|  |  |  |  |
| --- | --- | --- | --- |
| void broad.core.datastructures.MatrixWithHeaders.writeGCTWithHeaders | ( | String | *save*, |
|  |  | Map< String, String > | *columnMapping* |
|  | ) |  | throws IOException |

|  |  |  |  |
| --- | --- | --- | --- |
| void broad.core.datastructures.MatrixWithHeaders.writeGMT | ( | String | *save*, |
|  |  | double | *fold* |
|  | ) |  | throws IOException |

Here is the call graph for this function:

|  |  |  |  |  |  |
| --- | --- | --- | --- | --- | --- |
| void broad.core.datastructures.MatrixWithHeaders.writeRowNamesToFile | ( | String | *outFile* | ) | throws IOException |

|  |  |  |  |  |
| --- | --- | --- | --- | --- |
| void broad.core.datastructures.MatrixWithHeaders.zColumnNormalize | ( |  | ) |  |

Here is the call graph for this function:

|  |  |  |  |  |
| --- | --- | --- | --- | --- |
| void broad.core.datastructures.MatrixWithHeaders.zRowNormalize | ( |  | ) |  |

Here is the call graph for this function:

|  |  |  |  |  |  |
| --- | --- | --- | --- | --- | --- |
| MatrixWithHeaders broad.core.datastructures.MatrixWithHeaders.zScoresByColumn | ( | List< String > | *controlRows* | ) |  |

Here is the call graph for this function:

Here is the caller graph for this function:

---

The documentation for this class was generated from the following file:

- java/broad/core/datastructures/MatrixWithHeaders.java


---

Generated on Fri Aug 15 2014 15:10:52 for ESAT by  

 1.8.7
